# Recent Advances in Biomechanical Characterization of Thoracic Aortic Aneurysms

**DOI:** 10.3389/fcvm.2020.00075

**Published:** 2020-05-12

**Authors:** Hannah L. Cebull, Vitaliy L. Rayz, Craig J. Goergen

**Affiliations:** ^1^Weldon School of Biomedical Engineering, Purdue University, West Lafayette, IN, United States; ^2^Purdue Center for Cancer Research, Purdue University, West Lafayette, IN, United States

**Keywords:** thoracic, aorta, aneurysm, computational, modeling, imaging, biomechanical

## Abstract

Thoracic aortic aneurysm (TAA) is a focal enlargement of the thoracic aorta, but the etiology of this disease is not fully understood. Previous work suggests that various genetic syndromes, congenital defects such as bicuspid aortic valve, hypertension, and age are associated with TAA formation. Though occurrence of TAAs is rare, they can be life-threatening when dissection or rupture occurs. Prevention of these adverse events often requires surgical intervention through full aortic root replacement or implantation of endovascular stent grafts. Currently, aneurysm diameters and expansion rates are used to determine if intervention is warranted. Unfortunately, this approach oversimplifies the complex aortopathy. Improving treatment of TAAs will likely require an increased understanding of the biological and biomechanical factors contributing to the disease. Past studies have substantially contributed to our knowledge of TAAs using various *ex vivo, in vivo*, and computational methods to biomechanically characterize the thoracic aorta. However, any singular approach typically focuses on only material properties of the aortic wall, intra-aneurysmal hemodynamics, or *in vivo* vessel dynamics, neglecting combinatorial factors that influence aneurysm development and progression. In this review, we briefly summarize the current understanding of TAA causes, treatment, and progression, before discussing recent advances in biomechanical studies of TAAs and possible future directions. We identify the need for comprehensive approaches that combine multiple characterization methods to study the mechanisms contributing to focal weakening and rupture. We hope this summary and analysis will inspire future studies leading to improved prediction of thoracic aneurysm progression and rupture, improving patient diagnoses and outcomes.

## Introduction

Thoracic aortic aneurysms (TAAs) are pathological dilations, often associated with genetic disorders or other factors leading to cellular changes, elastic fiber degradation, collagen deposition, and inflammation (1, 2). TAA growth can lead to increased risk of dissection or complete rupture. Accurately defining these incidence rates remains challenging because the disease is often clinically silent until rupture or dissection occurs (3, 4) However, the number of TAA diagnoses are increasing due to improvements in medical imaging and screening with roughly 1 per 100,000 people affected (5). Approximately 60% of TAAs develop in the ascending thoracic aorta, while 40% occur in the descending portion (5, 6). Dissection frequently develops in the ascending portion of the thoracic aorta, resulting in a false lumen that can greatly increase rupture risk and has a mortality rate of up to 97% (7).

Current clinical guidelines suggest surgical intervention for TAAs once the vessel reaches 5–5.5 cm or a growth rate of >0.5 cm/year (2, 8). These guidelines are based on assessment of the risk of an intervention relative to that of dissection or rupture, with studies finding that faster growth increases the risk of rupture regardless of aortic diameter (3, 9). Guidelines may also differ slightly in patients with known genetic disorders (10). However, using diameter and expansion rates oversimplify the complexity of TAAs by neglecting body size, heterogenous wall composition, and hemodynamic loading of the vessel (11–14). Up to 50% of ascending thoracic dissections occur in vessels with diameters below the surgical intervention threshold, further suggesting these criteria oversimplify TAA pathologies (15–17).

Most TAA patients will be monitored with imaging and receive no treatment. The current estimated perioperative mortality rate of ascending aorta replacements is 3.4%, unless emergency replacement is required due to rupture or dissection, which has a much higher mortality of 15–24% (18, 19). To improve treatment, the relationship between TAA development and cellular composition, cellular responses, matrix remodeling, hemodynamics, vessel wall mechanics, and their relation to each other are currently being studied. Previous reviews have included a clinical perspective of TAAs (18), covered multiple types of aortic aneurysms (20), and focused on computational models (4), mechanotransduction (21), or genetics (10, 22). It is important to note that biomechanics of abdominal aortic aneurysms (AAA) have been more extensively investigated compared to TAAs, likely because AAAs have a higher incidence rate (4, 23, 24). In this review we present advances in the biomechanical characterization of human and animal TAAs including *ex vivo* and *in vivo* mechanical analyses, and modeling of blood flow and the vessel wall. Understanding TAA biomechanics represents a fundamental step in identifying underlying causes of growth and development that has the potential to improve patient outcomes (21).

## Risk Factors for TAA Development

TAAs are a multifactorial disease with risk factors including genetics, congenital defects, hypertension, smoking, and aging. Currently, there are 29 identified genes associated with TAA development, and efforts are underway to identify more (Supplementary Table 1) (10, 17, 25). Genetic disorders with increased risk include Ehlers-Danlos Syndrome, Loeys-Dietz Syndrome, Turner Syndrome, polycystic kidney disease, Alagille Syndrome and, the most commonly associated, Marfan Syndrome (MFS), where patients may even receive prophylactic surgery to prevent TAA formation (22, 26, 27). Bicuspid aortic valve (BAV), the most common congenital heart defect, occurs when two of the three valve cusps fail to separate, often leading to valve dysfunction. Approximately half of BAV patients develop stiffening of the ascending aortas, aneurysms, or dissections (28–30). Recent work shows that these aneurysms often develop as a result of genetic mutations or abnormal hemodynamic forces caused by BAV, highlighting the importance of investigating multiple factors in TAA formation (31–33).

In some cases, hypertension may play a role in TAA development and dissections due to high wall stresses (5, 31, 34–37). While age also plays a role in TAA development, it is difficult to isolate its influence because the human aorta naturally dilates about 0.15 mm/year, making it difficult to distinguish aneurysmal from healthy aortas (38, 39). In an attempt to quantify the role of age, an *ex vivo* study found that an increase in age caused a significant reduction in vessel wall strength, but no significant strength differences between age-matched aneurysmal and healthy tissue were observed (40). These findings suggest that age affects vessel strength regardless of disease severity.

## Pathophysiology of TAAs

Considering the complex structure and function of the vessel at the cellular level can help elucidate TAA biomechanics. The intima, a single layer of endothelial cells, communicates with the medial layer within the vessel. The media, composed of elastin lamellae and smooth muscle cells (SMCs), provides elasticity, while the primarily collagen-composed adventitia provides tensile strength (41–43). Together, these layers create a dynamic vessel capable of withstanding large hemodynamic forces. Though not fully understood, recent work has been made to develop novel theories of aneurysm development, predictive metrics for wall failure, and possible strategies for inhibiting growth and eventual rupture. SMCs play a significant role as they contribute to the vessel wall structure by producing ECM proteins including collagen, elastin, and laminin (44, 45). In the media of aneurysms, SMCs clonally expand and change to more phagocytic-like phenotypes (44, 46). Many studies have focused on the relationship between SMC death and TAA formation, but the relationship between the two remains unclear. One study found elevated apoptosis markers in aneurysmal vs. non-aneurysmal tissue (45), while other studies found no differences in SMC density or apoptosis in TAA tissue (44, 47). A possible clue could be from work reporting changes in SMCs from contractile to synthetic phenotypes, a transition that influences aneurysm formation by altering the balance between metalloproteinases (MMPs) and tissue inhibitors of MMPs (TIMPs) (44). MMPs are proteolytic enzymes released within the wall by SMCs that break down structural components including collagen, elastin, and SMCs, while TIMPs help control breakdown. During aneurysm progression, MMP activity increases while TIMPs decrease, causing an imbalance and further aortic wall degradation and dilation (26, 44). While these studies support the idea that change in SMC phenotype contributes to this imbalance, the cause of this transition remains unclear.

Various genetic disorders are associated with development of TAAs (10, 27, 48), but further investigation is needed to understand the role of genes in aortic wall behavior. The *FBN1* gene is commonly studied because of its role encoding the large glycoprotein, fibrillin-1, and mutations of this gene are found in MFS patients. It is well reported that TGFβ binds to proteins in the ECM, including fibrillin-1 (49, 50). Alterations in *FBN1* are correlated with reduced binding in MFS patients, leading to the release of TGFβ (49), ultimately affecting ECM structure and wall behavior (51, 52). Many other genes associated with TAAs have also been discovered, with ongoing research uncovering even more in order to identify possible therapeutic agents (50, 53–60).

Still, many TAA patients have no known genetic disorders, thus efforts should also be focused on biomechanics at both micro- and macro-scales. Mechanical differences may also contribute to wall composition changes as less elastin content is found in the aortic wall of BAV patients compared to TAV patients requiring valve replacements (61). Humphrey et al. extensively reviewed growth and remodeling of aneurysms and concluded that mechanosensing and ECM regulation are critical for maintaining mechanical homeostasis and proper function (41, 62). The ECM bears loads of up to 200 kPa, while still allowing the cells to sense mechanical stresses enough to respond with their proper functions (1, 21, 30, 41, 62–64). The theory that mechanosensing defects may cause aneurysms is based on the idea that if SMC function is impaired and the ECM loses organization, the cells may no longer function properly, causing wall expansion and failure. More comprehensive studies considering all of these possible causes of TAA formation need to be conducted to improve our knowledge of the biological, chemical, and mechanical influences on aneurysms.

## Animal Models

To gain a more fundamental understanding of TAAs, animal models are used because of the ability to track lesion progression and collect tissue without being limited by sample size or longitudinal image data. Further, animal studies allow greater control of physiologic variables such as diet, age, and genetic background. Given that *in vivo* cyclic strain is similar across species, the use of animal models is helpful for a variety of preclinical research (30, 65). Studies often use porcine models to quantify tensile testing of the thoracic aorta (66–70), while a wide range of TAA rodent models have been developed to study aneurysm development and progression (1, 37, 71–74). Over the past several decades, various TAA murine models have been established. Because of evidence showing a strong association between TAA development and genetic predisposition, murine models with genetic deficiencies have been created (1, 74, 75). Hypertensive models were also developed, including the commonly studied angiotensin-II model (31, 34, 36, 37).

**Figure 1:**
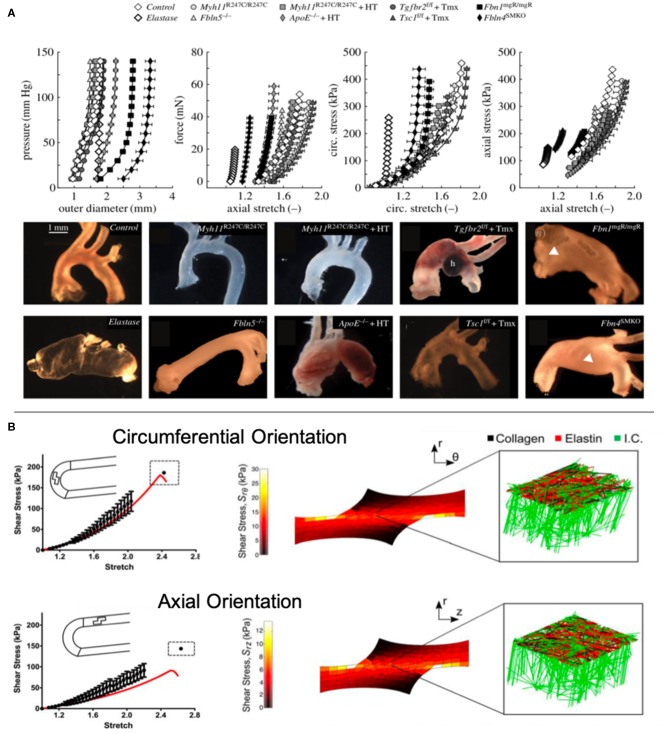
**(A)** Bellini et al. conducted *ex vivo* mechanical testing on ten mouse models to elucidate the biomechanical differences between models (1). **(B)** Witzenburg et al. used *ex vivo* testing to measure the effects of shear in the aortic wall and modeled the wall components (70).

Some animal studies focusing on the effects of TGFβ antibodies found that their administration neutralizes TGFβ activity (50), reducing dilation of the ascending aorta and risk of rupture (76). In a recent study, Bellini et al. conducted *ex vivo* biaxial testing to measure wall stresses, stiffness, and energy storage of ten murine models (8 TAA, 1 elastase positive control, 1 healthy negative control) to identify differences between models in a controlled setting. From their findings, the authors suggest that even with normal or reduced wall stresses, the intramural cells in the aneurysmal aortae could not maintain circumferential material stiffness, similar to the elastase control aorta. However, they found that structural stiffness may not be related to aneurysm formation finding decreased structural stiffness in both aneurysmal and non-aneurysmal vessels as revealed by their distensibility measurements (Figure 1A) (1). Their findings also demonstrate the importance of studying multiple models to increase translational potential as there is no exact phenocopy of human TAAs.

Using porcine models has some benefits including sharing similar structure, thickness, and composition to human aortae (Supplementary Table 1) (67). However, some studies have identified the need for porcine models that better represent the stiffness of aged (>60 years) or atherosclerotic human tissue by using aged animals or developing methods of artificially stiffening the aorta (77, 78). Further limitations of animal models include differences in size between small animals and humans, typical vessel curvature, and basal metabolic rate. Despite limitations associated with TAA animal models, they do provide an opportunity for consistent comparison between groups and studies, larger sample sizes, earlier stage tissues, and an ability to manipulate genomes.

## Biomechanical *ex vivo* Analysis

Mechanically testing aortic tissue provides insight into tissue deformation and failure. Common workflow for mechanical testing of tissues includes: (1) harvesting tissue, (2) cutting samples into desired shape, (3) preconditioning tissue in the testing machine under physiologic conditions, and (4) performing mechanical tests and analyses. With this approach, stress-strain data can be obtained and fitted to a strain energy function to help describe tissue behavior. The 2nd Piola-Kirchoff stress tensor and Green-Lagrange strain tensor in cylindrical coordinates are commonly used due to the large tissue deformations, non-linear material properties, and cylindrical vessel shape (66, 70, 79).

*Tensile testing*. Uniaxial and biaxial tensile tests can be performed on thoracic aortic tissue to determine behavior in circumferential and axial directions. Uniaxial testing measures tissue behavior in one direction, while biaxial allows for simultaneous tensile testing in two directions (30). Among biaxial tests is pressure-inflation testing which better mimics *in vivo* loading (Supplementary Table 1) (68). Of note, these tests have enabled researchers to assess differences in mechanical behavior between age, axial and circumferential location, orientation, aortic layers, and pathologies. The most common finding among these studies was increased stiffness and strength in the circumferential direction compared to axial (69, 80, 81), although two studies noted no significant differences between directions (82, 83). Histological findings reveal that collagen fibers tend to be aligned in the circumferential direction, suggesting that collagen plays a significant role in tissue strength (84). One study suggested that tissue failure may be correlated with collagen fiber, noting that localized collagen fiber bundles break during tensile testing (85). Recent studies have also considered the role of radial and shear forces in tissue failure (70, 86). Witzenburg et al. found that porcine thoracic aortas displayed failure at significantly lower shear stresses during lap tests compared to uniaxial and simulated their tests using a computational model with collagen and elastin components (Figure 1B) (70). This combined *ex vivo* testing and computational approach provided insight into the role that intramural stresses play on the growth and formation of TAAs.

*Tissue energy storage*. The aorta uses the energy it stores from expansion during systole to help deliver blood to the rest of the body, thereby making energy storage a critical parameter (18). In tensile testing, the energy loss of the loading cycles can be measured, where increasing loss indicates problems in aortic function (13). A study focusing on the role of elastin in energy storage of murine thoracic aortas found that only properly crosslinked elastin fibers were effective at storing energy (87). Yet, energy storage may not be a clear indicator of potential TAA rupture risk, as shown by another study of multiple murine TAA models that found comparable energy storage between diseased and control mice (88). This finding supports the idea that TAA characterization cannot rely on any one metric, but requires a comprehensive analysis including the effects of heart movement and surrounding structures on the thoracic aorta (89).

Though *ex vivo* data can provide extensive information about mechanical behavior of the tissue and rupture properties, it neglects respiratory, cardiac, and surrounding tissue motion which contribute to aortic motion. In addition, material testing can only be performed at one time point making it difficult to study TAA progression. Nonetheless, *ex vivo* mechanical testing provides important insight into mechanical properties, which can be combined with *in vivo* testing, histological analysis, and used in computational modeling.

## *In vivo* Imaging Analysis

Non-invasive imaging of TAAs has many benefits, including longitudinal studies and measuring aortic motion *in vivo* while posing minimal risk to the subject. Medical imaging is used clinically to measure diameters and expansion rates, while many studies have extended the application to include mechanical assessment of TAAs.

*Ultrasound*. Widely used in both clinical and research settings, ultrasound (US) offers minimal risk, portability, fast acquisition times, and low costs. However, the location of the thoracic aorta makes it difficult to acquire transcutaneous US without sternum and rib artifacts. When this occurs, a more invasive and time-intensive transesophageal US can be performed (20). Studies have used US to obtain TAA diameter measurements via motion-mode (M-mode) and time-resolved blood flow velocities using pulse wave velocity (PWV) or color Doppler modes. US is also used to quantify wall deformation and vessel geometries. Recent studies have used two-dimensional speckle tracking (2D-ST) techniques to measure *in vivo* strain of TAAs (90–93). Automatic aortic wall tracings are created from US data and manually optimized, providing estimates of regional and global wall deformations (93). One study used 2D-ST to calculate aortic stiffness and validated their measurements with biaxial tensile testing,^4^ highlighting that stiffness values can be estimated in a minimally invasive way. Other studies have used 2D-ST to validate results for simulated aortic biomechanics, finding good agreement (94, 95). While 2D approaches have been shown to be clinically relevant, they neglect regional differences caused by surrounding structures and heterogenous wall composition (96). 4DUS provides a way to estimate 3D strain by collecting time-resolved 3D images. Although this technique has not been directly applied to TAAs, recent studies have used 4DUS to characterize strain in animal and human abdominal aortic aneurysms (97, 98). Since aortic wall stress cannot be estimated with strain alone, it is important to supplement strain data by considering material properties. Therefore, combining 4DUS with *ex vivo* testing could help improve study significance and lead to an important clinical tool for improving patient stratification (18). In addition, data collected from 4DUS may be used to improve computational modeling as more hemodynamic models incorporate deformable walls (72, 79).

**Figure 2:**
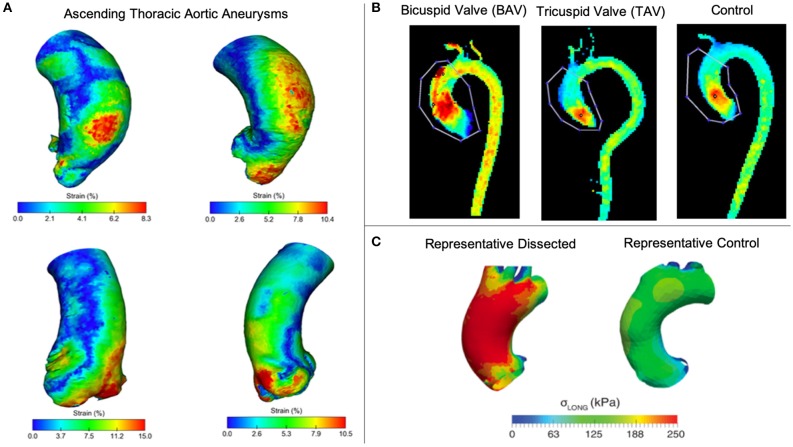
**(A)** Pasta et al. used gated CT to investigate 3D strain in TAAs (102). **(B)** Rahman et al. compared peak blood flow velocities between enlarged ascending thoracic aortas (TAV vs BAV) and a control (103). **(C)** Emerel et al. estimated wall stresses in dissected TAAs and compared results normal thoracic aortas (104).

*Computed Tomography*. Contrast-enhanced computed tomography (CT) is another effective method of assessing TAAs for surgical treatment based on high spatial resolution and larger field of view compared to US (20, 99). Many studies adopt a multi-modality approach using CT for defining patient-specific geometries, while using other approaches to quantify blood flow velocity, blood pressure, and wall deformation (14, 25, 100). That said, ECG-gated CT imaging data have recently been used to overcome the lack of wall deformation information (77, 101, 102). Pasta et al. collected gated CT data throughout the cardiac cycle (25 = TAA, 7 = non-TAA) and demonstrated the speed of this technique while providing full-field distribution of aortic wall strain (Figure 2A) (102), suggesting potential to develop a quick and reliable method for visualizing the full strain field of the thoracic aorta. Another study using ECG-gated CT estimated strain within the aortic wall but focused on the variations in aortic distensibility between locations and patients. Not surprisingly, the authors found that the ascending aorta had larger deformations than other parts of the vasculature (77). Limitations of this approach include additional exposure to ionizing radiation due to increased acquisition time and challenges in identifying and quantifying aortic wall thickness due to limited soft tissue contrast (99).

*Magnetic Resonance*. Magnetic resonance (MR) imaging offers a non-invasive method with great soft tissue contrast without using ionizing radiation (20). MR is prevalent in TAA research with growing interest on techniques for quantifying vessel deformation and blood flow including time-resolved 3D phase-contrast MR (4D flow MRI) (100, 105–107). 4D flow MR is used to measure blood flow velocities in 3D through the cardiac cycle. This approach is valuable in the thoracic aorta where high velocities, tortuous geometry, and the aortic valve all contribute to complex flow patterns (108). Schäfer et al. used 4D flow MR to investigate local hemodynamics in patients with chronic obstructive pulmonary disease. They found that reduced wall shear stress (WSS) and associated aortic stiffness in the mid-ascending aorta may contribute to the development of aneurysms or dissections (109). Other studies have used this technique to demonstrate that genetic disorders, including BAV and MFS, significantly alter flow patterns and velocity (11, 33, 110, 111). Figure 2B shows a comparison of peak velocity from 4D flow measurements between TAV and BAV patients with dilated ascending aortas and a control patient, finding the highest velocities in BAV patients (103). Cine-MR uses cardiac gating to collect dynamic information of vessel walls throughout the cardiac cycle. Studies use cine-MR to investigate wall deformation and displacement, with results demonstrating complex 3D movement of the vessel (105, 106). Because wall deformation and displacement affect the size of the landing zone for endovascular stents, cine-MR has potential use for surgical planning to reduce risk of endoleaks. In addition to using 4DUS or gated CT, cine-MR also can be used to estimate regional strain in the thoracic aorta as Wilson et al. demonstrates (107).

While imaging modalities can reveal regional heterogeneity in aortic wall strain, discerning the cause of regional variations is often not possible due to limited spatiotemporal resolution (Supplementary Table 2). Further, estimating wall thickness remains a challenge for all imaging modalities (24), and using *in vivo* imaging for characterizing vessel biomechanics does not allow for simulating hypothetical changes to the aortic wall or lumen. Comprehensive studies combining *in vivo* imaging with histological or *ex vivo* findings and computational simulations have potential to overcome these limitations (108, 112).

## Solid Mechanics

Numerical models incorporating tissue growth and remodeling are important for simulating aneurysm progression. Previously, models were frequently used to calculate stress within the vessel neglecting growth and remodeling (4, 113, 114). These static models oversimplify complex vessel geometry but can provide an initial step toward patient-specific growth and remodeling simulations. Pasta et al. combined tensile testing data with microstructural data obtained from multi-photon imaging to create a fiber-reinforced constitutive model for ascending TAAs, building upon their static constitutive model. They found that incorporating collagen fibers within their model indicated which ascending TAAs were prone to rupture or dissection based on wall stress values (115). Xuan et al. used solid mechanical modeling to study the effects of BAV and found that wall stress increases in both circumferential and longitudinal directions compared to patients with TAV (116). These findings confirm previous *ex vivo* studies (117, 118), as well as a recent study that found greater degeneration in TAV vs. BAV aneurysmal aortic tissue (119). The authors also attempted to correlate ascending TAA diameter and peak wall stress in order to predict dissection risk. While higher wall stresses were found in BAV models, the higher stresses did not correlate to larger diameters as were found in TAV models, confirming that diameters alone are insufficient to predict dissection and wall stresses should be examined further. In another study, Emerel et al. found that wall stress significantly increased only in the longitudinal direction for patients with TAA dissections (Figure 2C) (104). It is important to note limitations associated with solid mechanics analyses of TAAs, such as the lack of residual stress or thickness heterogeneity, no inclusion of elastin degradation, neglecting effects of momentum and shear, and using isotropic vs. anisotropic constitutive laws (4, 113, 115, 120). As a result, future work could incorporate anisotropic constitutive laws and vascular remodeling.

## Computational Fluid Dynamics and Fluid-Structure Interactions

Computational fluid dynamics (CFD) simulates blood flow in various cardiovascular environments in order to quantify relevant hemodynamic metrics, e.g., velocity, pressure and wall shear stress (WSS). A main advantage of CFD is the ability to simulate blood flow in complex subject-specific geometries, providing results with high spatial and temporal resolution (108). Fluid-structure interaction (FSI) is a type of CFD that provides a more advanced approach accounting for wall deformation. In FSI simulations, the coupled fluid and solid mechanics equations are solved using a two-way coupling method between the domains or using an approach where the fluid and solid equations are solved together by the same solver known as the Monolithic approach (109, 121, 122) We highlight studies using both rigid and deformable vessel walls below.

CFD studies can quantify subject-specific distributions of relevant flow metrics (11, 12, 123–128). For example, CFD studies conducted for BAV patients revealed increased flow asymmetry, residence time, and WSS in the aortic arch, thus increasing the potential for TAA development (128). A recent study focused on vessel size and morphology, found that diameters enlarged more than 60% relative to baseline resulted in significantly altered blood flow patterns, including impinged flow near the expansion (123). A similar study investigated the effects of distal branches on TAAs by simulating blood flow in a bovine arch, quantifying the relationship between WSS values and rupture; however, no correlation was observed between increased WSS and decreased tissue strength (124). Phillips et al. also investigated geometry-specific variations by simulating flow through murine aortic dissections, concluding that varying thickness and lesion compositions found in their histological samples were due to variations in false lumen flow and vortical structures (125). Acuña et al. extensively reviews CFD in animal models in a broad range of vascular diseases (129). Taken together, these CFD studies demonstrate that small geometrical variations may cause large hemodynamic changes, greatly influencing vessel wall stresses and progression of TAAs.

FSI studies are often used to improve the assessment of WSS in image-based geometries, by incorporating pulsatile motion of deformable vessel walls and the aortic valve using various mechanical models (95, 122, 130–132). Yeh et al. studied WSS distributions in three patients by creating FSI models of ascending TAAs with anisotropic hyperelastic material properties (79). Although only a limited section of an idealized ascending TAA was modeled, the authors suggest that changing blood pressure levels caused varying maximum WSS between patients despite minimal differences in velocity, suggesting that the changes in WSS were geometry-dependent (79). Cao et al. used simulations to investigate the effects of aortic valve geometries on WSS in the aortic arch by comparing TAV to the various forms of BAV (133). Results showed that WSS directionality was significantly affected by BAV morphologies, indicating this computational approach may help determine aortopathy prognoses for BAV patients. Another study, focused on effects of hypertension and wall stiffness, found that stiffer TAAs also had the highest peak wall stresses (130), agreeing with results from a similar CFD study (12). As the field advances, use of FSI modeling of the thoracic aorta will likely increase because the results demonstrate that large vessel deformations affect relevant hemodynamic metrics.

While useful in many aspects, it is important to recognize the limitations associated with computational modeling. Studies using rigid walls neglect the effects of the deforming vessel wall on hemodynamics and inherent uncertainty in the model inputs, including inlet and outlet flow conditions (24). While FSI does consider vessel wall deformation, it is challenging to accurately model wall thickness, residual stress, and material properties; since these metrics are hard to obtain *in vivo*, these values are often based on previous literature (130, 131). Moreover, the collection of reliable *in vivo* data used for prescribing boundary conditions also remains a challenge for both rigid and deformable wall simulations (20). Although simulations will always have certain limitations, hemodynamic modeling has the potential to advance our understanding of TAA disease progression and rupture risk.

## Conclusions and Future Directions

The biomechanical analyses briefly presented in this review illustrate our current understanding of TAA development and progression. Literature supports the view that diameter and expansion rates are insufficient for determining rupture risk. The diverse etiologies have varying vessel geometries, rates of elastin degradation, collagen turnover, cellular changes, and inflammation, all contributing to the heterogeneity of thoracic aorta wall motion and integrity. To characterize these aspects, studies use *in vivo, ex vivo*, and *in silico* methods to estimate wall deformation, strain, wall shear stresses, and stiffness. Many of the studies summarized in this review focused on just one of these methods or properties in an attempt to find a correlation to aneurysm development. However, inherent limitations with each individual method suggests use of a comprehensive approach that combines these modalities to yield increasingly impactful results. Moreover, using characterization methods that consider the heterogeneity of TAAs is critical for understanding how the behavior relates to the structural composition of the aneurysms. By conducting interdisciplinary studies that consider the 3D heterogeneity, we can move toward a more complete understanding of TAAs and how to properly treat them, ultimately improving patient outcomes.

## Author Contributions

HC wrote the text of this review paper with guidance from CG and VR. All authors have reviewed the final version and approve of the content in this manuscript.

## Conflict of Interest

The authors declare that the research was conducted in the absence of any commercial or financial relationships that could be construed as a potential conflict of interest.
