# Supplementary material for: Recent Advances in Biomechanical Characterization of Thoracic Aortic Aneurysms
Source: Front Cardiovasc Med. 2020 May 12;7:75. doi: 10.3389/fcvm.2020.00075 (PMC7235347; doi:10.3389/fcvm.2020.00075)
Supplement: Supplementary file 1 [file Data_Sheet_1.docx]

Supplementary Material

| **Supplementary Table 1.** Summary of major findings from recent *ex vivo* studies presented in chronological order. | | | | |
| --- | --- | --- | --- | --- |
| Author | Test Type | Sample | Pathology | Major Findings |
| Sokolis et al. (2011)^81^ | Uniaxial | Human | 14 non-TAA 12 ATAA | Tissue in circumferential is stronger and stiffer vs. axial direction |
| Khanafer et al. (2011)^134^ | Uniaxial | Human | 13 ATAA | Tissue in circumferential is stronger and stiffer vs. axial direction |
| Kim & Baek (2011)^68^ | Inflation | Porcine | 7 non-TAA | Tissue in posterior region is stiffer and thinner vs. anterior |
| Sokolis et al. (2012)^135^ | Uniaxial | Human | 8 ATAA | Medial and adventitial tissue were stiffer in circumferential direction, but intimal showed no significant difference |
| García-Herrera  et al. (2012)^40^ | Uniaxial | Human | 23 non-TAA 14 ATAA 12 BAV | Aging causes reduction in strength, but no significant differences found in strength between pathologies and non-TAA tissue |
| Labrosse et al. (2012)^136^ | Inflation | Human | 24 non-TAA | Developed material constants for finite element modeling categorized by age, gender, and location |
| Martin et al. (2013)^137^ | Biaxial Uniaxial | Human | 20 ATAA 17 BAV 13 BAA | Decreasing tissue compliance directly related to increasing chance of rupture |
| Azadani et al. (2013)^82^ | Biaxial | Human | 19 non-TAA 18 ATAA | ATAA tissue is stiffer vs. non-TAA tissue; no stiffness difference in circumferential vs. axial direction |
| Pham et al. (2013)^80^ | Biaxial | Human | 20 ATAA 20 BAV 15 BAA | Tissue in circumferential is stiffer vs. axial direction |
| Shah et al. (2014)^69^ | Biaxial Uniaxial | Porcine | 11 non-TAA | Tissue in circumferential is stronger vs. axial direction |
| Pierce et al. (2014)^83^ | Uniaxial | Human | 7 TAA | Isotropic behavior is based on fiber orientation; no stiffness difference in circumferential vs. axial direction |
| Forsell et al. (2014)^115^ | Uniaxial | Human | 11 TAA 13 BAV | BAV thoracic tissue is stronger and stiffer vs. TAV tissue |
| Trabelsi et al. (2015)^138^ | Inflation | Human | 5 ATAA | Highlighted variations between patients including a large range of peak wall shear stress values; developed a rupture-risk assessment |
| Pasta et al. (2016)^113^ | Uniaxial | Human | 4 non-TAA 6 ATAA 8 BAV | ATAA have altered collagen fiber alignment vs. non-TAAs; fiber distribution influences wall stress more than peak stress |
| Smoljkić et al. (2016)^139^ | Biaxial Uniaxial | Human | 2 ATAA 4 BAV | Found large variability in wall thickness, therefore highlighting the inaccuracies in estimating wall stress |
| Sommer et al. (2016)^86^ | Uniaxial Triaxial Shear | Human | 9 ATAA 3 CTD 4 DIS | Anisotropic failure properties and higher shear in axial vs. circumferential; DIS tissue showed lower strength vs. TAA |
| Davis et al. (2016)^140^ | Inflation | Human | 6 ATAA | Found increasing stiffness with increasing age; discovered substantial strain heterogeneity from regional to local areas |
| Duprey et al. (2016)^84^ | Uniaxial Inflation | Human | 23 ATAA 8 BAV | Tissue in circumferential is stiffer and stronger vs. axial direction; collagen in media aligns in circumferential direction |
| Peña et al. (2017)^141^ | Biaxial | Porcine | 7 non-TAA | Abdominal aorta is stiffer and more anisotropic vs. thoracic; confirmed higher stiffness in circumferential vs. axial direction |
| Witzenburg et al. (2017)^70^ | Uniaxial Biaxial Lap Shear | Porcine | 6 non-TAA | Higher stiffness and strength in circum. vs. axial direction; failure occurs at lower shear stresses vs. circum. and axial |
| Acosta-Santamaria et al. (2018)^66^ | Uniaxial | Porcine | 1 non-TAA | Uniaxially tested across whole thickness using OCT; tracked the variations in thickness for each layer under tension |
| Thunes et al. (2018)^85^ | Uniaxial | Human | 7 non-TAA 13 TAA 23 BAV | Studied relationship between collagen and intimal tears; found collagen breakage may be the governing tissue failure mechanism |
| Abbreviations: ATAA = ascending thoracic aortic aneurysm; BAV = bicuspid aortic valve; CTD = connective tissue disorder; DIS = dissection; OCT = optical coherence tomography; TAA = thoracic aortic aneurysm, TAV = tricuspid aortic valve | | | | |

| **Supplementary Table 2.** Comparison of image modalities used to characterize dynamic vessels in humans. Range of resolution values presented are based on previous studies. | | |  |
| --- | --- | --- | --- |
| Image  Modality | Temporal Resolution | Spatial  Resolution |  |
| 4D Clinical Ultrasound^20,142^  (3 – 7.5 MHz) | 5 – 24 ms | Axial: 0.2 – 3 mm  Lateral: 0.2 – 3 mm |  |
| ECG-Gated CT^9,102^ | 85 – 135 ms | Axial: 0.5 – 0.625 mm  Lateral: 0.5 mm |  |
| Cine-MR^9,103,105^ | 20 – 50 ms | Axial: 0.8 – 8 mm  Lateral: 0.75 – 1.3 mm |  |
| Abbreviations: CT = computed tomography; Cine-MR = Cine-magnetic resonance imaging | | |  |

**REFERENCES**

134. Khanafer K, Duprey A, Zainal M, Schlicht M, Williams D, Berguer R. Determination of the elastic modulus of ascending thoracic aortic aneurysm at different ranges of pressure using uniaxial tensile testing. *J Thorac Cardiovasc Surg*. (2011) 142:682–86. doi: 10.1016/j.jtcvs.2010.09.068

135. Sokolis DP, Kritharis EP, Iliopoulos DC. Effect of layer heterogeneity on the biomechanical properties of ascending thoracic aortic aneurysms. *Med Biol Eng Comput*. (2012) 50:1227–37. doi: 10.1007/s11517-012-0949-x

136. Labrosse MR, Gerson ER, Veinot JP, Beller CJ. Mechanical characterization of human aortas from pressurization testing and a paradigm shift for circumferential residual stress. *J Mech Behav Biomed Mater.* (2012) 17:44–55. doi: 10.1016/j.jmbbm.2012.08.004

137. Martin C, SunW, Pham T, Elefteriades JA. Predictive biomechanical analysis of ascending aortic aneurysm rupture potential. *Acta Biomater*. (2013) 9:9392–400. doi: 10.1016/j.actbio.2013.07.044

138. TrabelsiO, Davis FM, Rodriguez-Matas JF, Duprey A, Avril S. Patient specific stress and rupture analysis of ascending thoracic aneurysms. *J Biomech*. (2015) 48:1836–43. doi: 10.1016/j.jbiomech.2015.04.035

139. Smoljkic M, Fehervary H, Van den Bergh P, Jorge-Peñas A, Kluyskens L, Dymarkowski S, et al. Biomechanical characterization of ascending aortic aneurysms. *Biomech Model Mechanobiol.* (2016) 16:705–20. doi: 10.1007/s10237-016-0848-4

140. Davis FM, Luo Y, Avril S, Duprey A, Lu J. Local mechanical properties of human ascending thoracic aneurysms. *J Mech Behav Biomed Mater*. (2016) 61:235–49. doi: 10.1016/j.jmbbm.2016.03.025

141. Peña JA, Corral V, Martínez MA, Peña E. Over length quantification of the multiaxial mechanical properties of the ascending, descending and abdominal aorta using digital image correlation. *J Mech Behav Biomed Mater*. (2017) 77:434–45. doi: 10.1016/j.jmbbm.2017.10.007

142. Huang Q, Zeng Z. Review article a review on real-time 3D ultrasound imaging technology. *Biomed Res Int Biomed Res Int*. (2017) 2017:1–20. doi: 10.1155/2017/6027029
